# Detection of genome‐edited and endogenously expressed G protein‐coupled receptors

**DOI:** 10.1111/febs.15729

**Published:** 2021-02-09

**Authors:** Mark Soave, Leigh A. Stoddart, Carl W. White, Laura E. Kilpatrick, Joëlle Goulding, Stephen J. Briddon, Stephen J. Hill

**Affiliations:** ^1^ Division of Physiology, Pharmacology and Neuroscience School of Life Sciences University of Nottingham UK; ^2^ Centre of Membrane Proteins and Receptors (COMPARE) University of Birmingham and University of Nottingham The Midlands UK; ^3^ Harry Perkins Institute of Medical Research and Centre for Medical Research QEII Medical Centre The University of Western Australia Nedlands Australia; ^4^ Australian Research Council Centre for Personalised Therapeutics Technologies Australia; ^5^ Division of Biomolecular Science and Medicinal Chemistry School of Pharmacy, Biodiscovery Institute University of Nottingham UK

**Keywords:** advanced imaging, CRISPR/Cas9, endogenous, fluorescent ligand, GPCRs, nanobodies

## Abstract

G protein‐coupled receptors (GPCRs) are the largest family of membrane receptors and major targets for FDA‐approved drugs. The ability to quantify GPCR expression and ligand binding characteristics in different cell types and tissues is therefore important for drug discovery. The advent of genome editing along with developments in fluorescent ligand design offers exciting new possibilities to probe GPCRs in their native environment. This review provides an overview of the recent technical advances employed to study the localisation and ligand binding characteristics of genome‐edited and endogenously expressed GPCRs.

## Graphical abstract

G protein‐coupled receptors (GPCRs) are the largest family of membrane receptors and major targets for FDA‐approved drugs. Quantifying GPCR expression and ligand binding characteristics in different cell types is important for drug discovery. The advent of genome editing and developments in fluorescent ligand design offers exciting possibilities to probe GPCRs in their native environment. This review discusses recent techniques employed in studying endogenously expressed GPCR localisation and ligand binding characteristics.

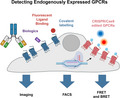

**Abbreviations**
BRET: bioluminescence resonance energy transfer
FCS: fluorescence correlation spectroscopy
GPCR: G protein‐coupled receptor
HILO: highly inclined and laminated optical sheet
PALM: photo‐activated light microscopy
PET: positron emission topography

## Introduction

G protein‐coupled receptors (GPCRs) are membrane proteins characterised by seven transmembrane‐spanning domains, an extracellular N terminus and an intracellular C terminus. They represent the single largest family of proteins targeted by FDA‐approved drugs; approximately 35% of all drugs target a GPCR [1]. GPCRs are widely expressed in all tissues; however, the expression pattern of individual receptor subtypes varies extensively between cell types [2]. It is partly this selectivity of expression by different cell types that helps account for the tissue‐selective actions of many GPCR‐targeted drugs. Individual GPCR subtypes are often natively expressed at low levels physiologically [3], and many GPCR subtypes can be up‐ or down‐regulated in disease states [1]. These factors make the study of cellular distribution and localisation of GPCRs in their native environment challenging.

**Fig. 1:**
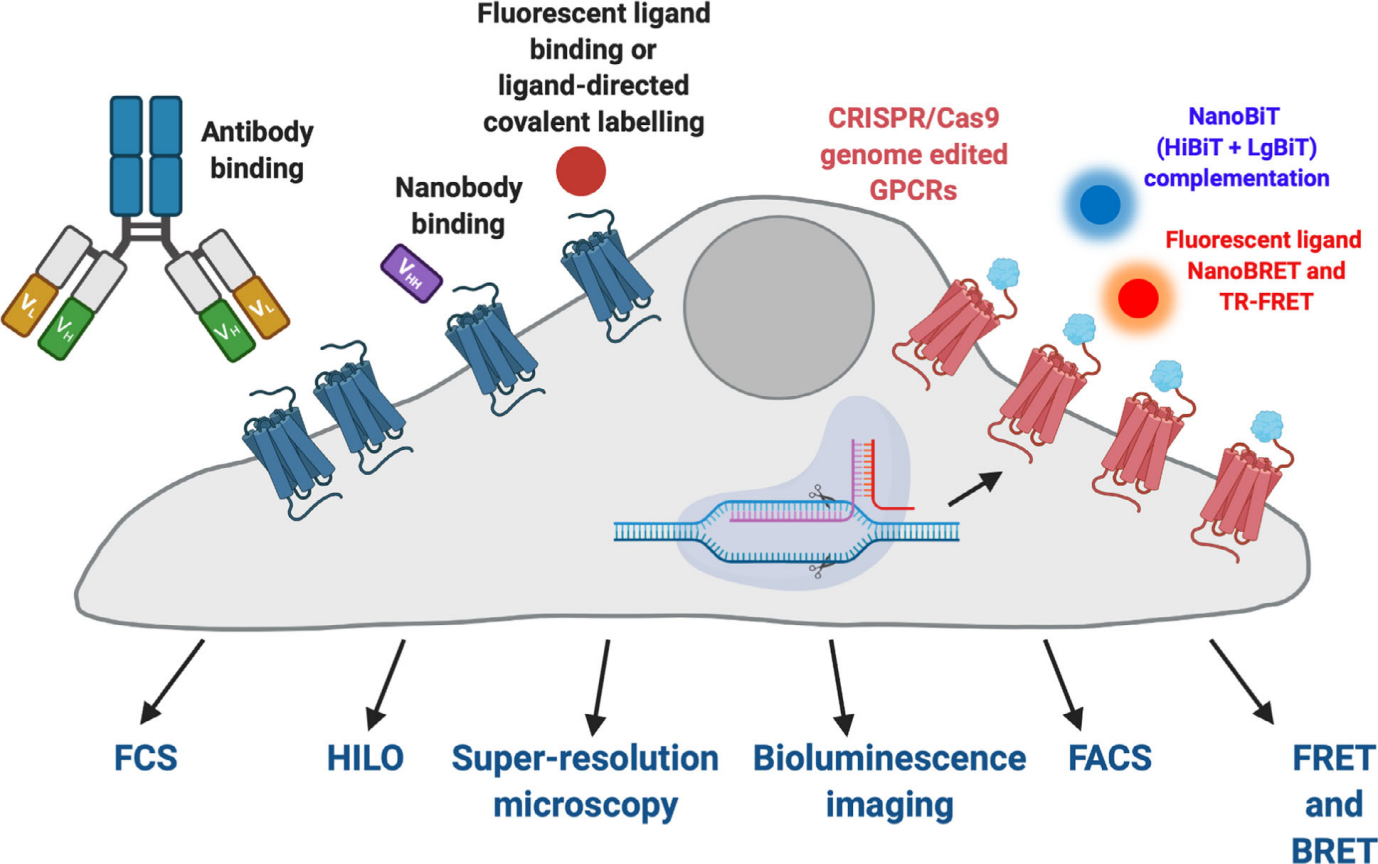
New approaches to tag and study endogenous GPCRs in living cells. These include the use of antibodies, nanobodies, fluorescent ligands and CRISPR/Cas9 genome editing in combination with confocal microscopy, bioluminescence microscopy, fluorescence correlation spectroscopy (FCS), bioluminescence (BRET) and time‐resolved Förster (TR‐FRET) resonance energy transfer, fluorescence‐activated cell sorting (FACS), highly inclined and laminated optical sheet (HILO) microscopy and the application of NanoBiT complementation. Figure prepared in ©BioRender (www.biorender.com).

There have been many studies using agonists and antagonists to probe the function of GPCRs in primary cells and tissues. However, these experiments rely on the expression profiling of the receptors in the primary tissue to clarify that the sample in question endogenously expresses the given receptor. To combat this, techniques have been developed to study GPCR populations at the endogenous level and in native tissues; these include novel biosensors and probes, progress in genetic engineering and advanced imaging techniques. This review will focus on new and emerging techniques (Table 1, Figure 1) to detect and understand the cellular spatiotemporal organisation of endogenously expressed GPCRs.

**Table 1:** Summary of techniques to detect endogenous GPCRs discussed in this review. Advantages and disadvantages of each technique are described, alongside examples.

Technique	Advantages	Disadvantages	Example GPCRs
Antibodies	Versatile: able to perform many different assays with the same anti‐GPCR antibody Can conjugate to fluorescent dyes or heavy metal ions for microscopy Retention *in vivo* achieved through antibody recycling	Not all GPCRs have specific subtype‐selective antibodies Expensive to generate antibodies against new targets	CGRP [24] CXCR4 [37] FPR2 [36]
Nanobodies	Easily genetically or chemically modified Can be purified from bacterial cultures in large quantities Improved tissue penetration compared to full‐length antibodies due to smaller size Can be conjugated to other proteins for improved binding characteristics or retention	Low retention *in vivo* due to glomerular filtration and excretion Could be challenging to find noncompetitive extracellular nanobodies for peptidergic receptors due to overlap between extracellular epitopes and the ligand binding site Few GPCRs have extracellular‐binding nanobodies	ACKR3 (CXCR7) [54] CXCR2 [53] CXCR4 [55, 56, 57, 58, 60, 62] PTH1R [52] US28 [65, 66]
CRISPR/Cas9	Simple and efficient modification of target receptor Can be used to append reporter tags (fluorescent, bioluminescent, self‐labelling or epitope) onto target receptor Can introduce disease‐relevant SNPs	GPCR is fused to a tag which may change its function or stability Potential for off‐target editing of the native genome Editing requires suitable location of a protospacer adjacent motif (PAM) Requires validation to ensure correct in‐frame editing of target Editing in non‐diploid cell lines may result in heterozygous inserts	ACKR3 [69] Adenosine A_2B_ receptor [71] β_2_‐adrenoceptor [68] CXCR4 [68, 69, 74, 75]
Fluorescent Ligands	Can visualise receptor localisation *in vitro* Can perform ligand binding assays via microscopy or via FRET/BRET donor emission	Requires a selective ligand Fluorescent ligand occupies ligand binding site, so functional effects are governed by the pharmacology of the probe	Adenosine A_3_ receptor [96] Cannabinoid CB_2_ [95] CXCR4 [94] Histamine H_1_ [87] TSHR [88]
Covalent ligand‐directed labelling	Noninvasive approach Labels the target receptor without affecting the ligand binding site Can be applied to monitor receptor trafficking and internalisation	Requires extensive ligand design to validate Target GPCR needs to contain suitable amino acids in close proximity to ligand binding site	Adenosine A_2A_ receptor [103, 109] Bradykinin B2 [107] Cannabinoid CB_2_ [104] µ opioid receptor [108]

## Challenges with studying endogenous GPCRs

The isolation of specific GPCR genes makes it possible to introduce human GPCRs into recombinant cells through transfection, allowing for the detailed study of specific GPCR subtypes in isolation. These experiments rely on the overexpression of GPCRs as models to study receptor organisation, ligand binding, and signalling. GPCRs are often expressed in cellular backgrounds which do not reflect their natural environment, and often at expression levels far exceeding those found physiologically. Furthermore, receptor overexpression results in a shift in the relative abundance of interacting partners, including G proteins, β‐arrestins, and potential dimer partners which are endogenously expressed in the recombinant cell. These parameters become important when considering phenomena such as receptor reserve, which can dramatically impact the signalling responses measured in a particular assay [4]. GPCR overexpression therefore removes the subtlety inherent in a physiological system.

To further add to the challenges of studying native GPCR expression, quantification of GPCR mRNA expression does not always correlate with the receptor expression observed at the cell surface [5]. Sriram *et al*. [5] compared the RNA expression of a panel of G_q_ coupled GPCRs to their expression at the cell surface, using increases in intracellular calcium to reflect receptor activation. Several high‐throughput RNA quantification techniques were studied, with single cell RNA‐seq proving to be the most reliable predictor. However, techniques with low sensitivity yielded many false negative results, indicating receptors which were expressed, but not detected by these high‐throughput assays. This becomes particularly challenging when accounting for the low endogenous expression of many GPCRs. The discrepancies between mRNA expression and protein expression aren't limited to GPCRs, as a recent study has shown a discordance between mRNA and protein enrichment patterns depending on the tissue being examined [6]. Additionally, the presence of previously unknown GPCR splice variants which may lead to altered ligand binding characteristics, or even nonfunctional receptors [7, 8], may lead to false positive results at the mRNA level. These results show that mRNA expression alone cannot account for the detection or quantification of endogenously expressed GPCRs, rather their detection requires quantification at the protein level.

Traditionally, radiolabelled ligands (radioligands) have been used extensively to study GPCR pharmacology, including in binding assays to determine the binding affinities and kinetic rate constants for both radioligands and unlabelled ligands [9]. Radioligands are ligands which have been modified to include a radioisotope, such as [^3^H], [^14^C], [^18^F] or [^125^I] [10]. The specifically bound radioactivity can then be monitored following filtration to separate bound and free radioligand or with the use of scintillation proximity beads, using a scintillation counter. Radioligands can therefore detect the presence of the receptor in question. Radioligands have also been used in combination with positron emission topography (PET) imaging to visualise and label specific GPCRs *in vitro* and *in vivo*, usually in the central nervous system [11, 12, 13]. This approach has been useful for detecting changes in receptor expression under disease conditions [12]. Additionally, a number of peptides have been used as radioligands to detect and target their endogenous receptor in diagnostic settings, such as for the neurotensin receptor 1 [14], and the somatostatin receptors [15]. However, not all GPCRs have radioligands, such as recently deorphanised GPCRs, or have radioligands with sufficiently high affinity to be practical in a radioligand binding experiment, such as with the β_3_‐adrenoceptor [16]. For these receptors, antibodies are often the primary means of detecting receptor expression.

Antibodies are glycoprotein heterodimers consisting of two identical heavy chains and two identical light chains linked by disulphide bonds [17]. The N‐terminal region of both heavy and light chains varies between different antibodies. This region constitutes the variable fragment which recognises the antigen. Antibodies are versatile tools which often bind to their target with high affinity. Through the use of labelled secondary antibodies, they can be used to detect the presence of a specific protein with a wide range of techniques, including immunohistochemistry, proximity ligation assays, immunoblotting, or colorimetric detection with an ELISA. GPCRs genetically modified with epitope tags (such as FLAG‐, HA‐ or His‐tags) can easily be detected using antibodies against the relevant tag. However, the generation of antibodies against unmodified GPCRs (including endogenously expressed GPCRs) has several significant hurdles to overcome, including low cell surface expression, the need for the GPCR to be expressed in a membrane with the correct post‐translational modifications, and the conformational heterogeneity of GPCRs [18]. Furthermore, GPCRs exhibit low immunogenicity, with only the N‐terminal domain and extracellular loops accessible as potential epitopes for extracellularly targeted antibodies. The generation of intracellular‐binding anti‐GPCR antibodies is useful for the detection of receptors in fixed and permeabilised samples, which would allow the antibody to access its cytoplasmic epitope, such as antibodies which bind specific phosphorylated residues on the C‐terminal tail of GPCRs [19]. However, these intracellular binders still require full characterisation, and suffer from the issues with low immunogenicity and antibody generation described above. With their larger N‐terminal extracellular regions, there has been some success generating functional antibodies targeted against chemokine GPCRs and class B GPCRs [20, 21, 22, 23]. This has resulted in the FDA approval of GPCR‐targeted antibody therapies for migraine, via targeting the CGRP receptor [24], and certain T‐cell lymphomas, via antibody targeting the chemokine CCR4 receptor [25].

Antibodies raised against GPCRs with small N‐terminal domains, such as the aminergic family of GPCRs, remain challenging to produce [26]. This has resulted in antibodies which lack specificity for their target, or do not recognise their target receptor in its naturally folded state [27]. Pradidarcheep *et al*. [28] tested antibodies raised against each of the five muscarinic and nine adrenoceptors. They found distinctive immunohistological staining patterns for each antibody, but significant nonspecific staining of whole‐cell extracts stably expressing only one receptor when using western blotting. As an example, antibodies against the α_2B_‐, β_2_‐ or β_3_‐adrenoceptors produced similar bands on their immunoblots, with significant binding observed in cell lines not expressing the target receptor. Similar results were seen comparing commercially available antibodies against the β_3_‐adrenoceptor [29]. Many anti‐GPCR antibodies also demonstrate batch‐to‐batch variations and thus, poor experimental reproducibility. As an example, Grimsey *et al*. [30] compared several commercially available antibodies against the N terminus of the cannabinoid CB_1_ receptor, and tested them with a wide range of immunological techniques. They found batch‐to‐batch variations in the degree of staining of the CB_1_ receptor in brain sections and when expressed in whole cells. These variations were compounded with poor species specificity and high cross‐reactivity of these antibodies when analysing cell lysates with western blots [30].

Issues with GPCR‐targeted antibodies can be tackled using several approaches. For example, the use of purified or thermostabilised receptor populations for immunisation, immunisation of the target receptor DNA directly into the host organism, or immunisation with peptide fragments have all yielded antibodies which can specifically bind their target GPCR [31, 32, 33]. These advances have led to the development of novel GPCR antibodies, providing new opportunities to visualise endogenous receptor localisation. However, it is important to consider species differences which may occur if the target receptor and the receptor used as the immunogenic stimulus originate from a different species. This is crucial as the extracellular domains (N terminus and three extracellular loops), which are the most immunogenic part of a GPCR, often show low homology between species [34].

Most biochemical approaches to detect GPCRs require the sample be immobilised through fixation which can introduce artefacts, permeabilised to allow intracellularly targeted antibodies to access their epitope, or denatured in harsh conditions by reducing agents prior to analysis on reducing gels. Although useful, these techniques only provide a snapshot of the receptor population which is frozen in time. The use of selective antibodies with flow cytometry has facilitated the real‐time detection of endogenously expressed cell surface proteins on a range of live cell types. Its wider use to study endogenously expressed GPCRs has been somewhat limited by the paucity of selective antibodies available, but has included the detection of the noncanonical GPCR Mas‐related G protein‐coupled receptor X2 (MRGPRX2) on human LAD2 mast cells [35], the formyl peptide receptor 2 (FPR2) on human chondrocytes [36], CXCR4 on Jurkat, T lymphoid SupT1 and MT‐4 T cells [37] and differential expression on different human leukocytes (T cells, monocytes, neutrophils, eosinophils, B cells and NK cells) of the dopamine receptor subtypes D2, D3, D4 and D5 [38]. The recent expansion in development of well validated selective tools to study GPCRs (e.g. fluorescent ligands, nanobodies) will allow flow cytometry to be more widely used to detect the expression of endogenous GPCRs in complex samples of heterogeneous expression (e.g. tumours, immune cells), in addition to allowing comparison to other cell surface protein expression, potential interactions of GPCRs with other proteins (cell surface and intracellular) and the study of single cell GPCR pharmacology in real time, to potentially reveal molecular mechanisms underlying health and disease. For example flow cytometry used in conjunction with fluorescence resonance energy transfer (FRET; FRET/FACS) has allowed the measurement of protein–protein interactions of the human and simian immunodeficiency virus (HIV and SIV) Vpu protein with the restriction factor CD137 (Bst‐2 or tetherin) that must be overcome to allow HIV‐1 release from infected cells [39]. Here, FRET/FACS had the advantage of combining the relative spatial sensitivity of FRET to measure protein–protein interactions with the much greater throughput possible with flow cytometry. The high throughput of flow cytometry has also allowed the detection and profiling of 363 cell surface antigens (largely cluster of differentiation markers) into biologically relevant clusters that have been used to classify a range of cell types including PBMCs, epithelial cancer cell lines, melanoma cell lines, leukaemia cell lines and cultured fibroblasts [40]. In the flow cytometry studies that have investigated disease‐relevant GPCRs, decreased GPR18 expression has been observed on polymorphonuclear neutrophils from sepsis patients when compared to healthy volunteers [41]. Flow cytometry measurements of dual‐immunolabelled arginine vasopressin receptor 1A (AVPR 1A) and atypical chemokine receptor 3 (ACKR3) expression in human vascular smooth muscle cells has also supported evidence that these receptors form heteromers that may underlie interactions between the innate immune and vasoactive neurohormonal systems [42].

Alternatively, antibodies can be radiolabelled and administered to detect GPCRs *in vivo*. At the time of writing, two GPCR‐targeting antibodies have been used to image GPCRs *in vivo*, both targeted against chemokine receptors. These antibodies were labelled with [^89^Zr] and were used to visualise CXCR4 or ACKR3 expression in murine xenograft tumours using PET imaging [43, 44]. Additionally, ACKR3 was visualised in these tumours with an [^125^I] radiolabelled antibody [44]. While these studies are impressive, the lower resolution of PET does not allow for the cellular localisation of the receptors to be studied.

## Single‐domain antibodies to interrogate endogenous GPCRs

In the last few years, single‐domain antibody fragments (sdAbs) have emerged as interesting tools to study GPCR localisation and pharmacology. These sdAbs are derived from the variable region of heavy chain‐only antibodies, which are found in camelids (nanobodies) or in cartilaginous fish (V_NARs_) [17, 45]. Nanobodies are small, being only 12–15 kDa in size as they lack the corresponding light chain found in conventional mammalian antibodies. They retain the high affinity binding which is characteristic of antibodies; but unlike full‐length mammalian antibodies, they require no post‐translational modification and can easily be purified in large quantities through expression in *Escherichia coli* [17]. Encoded by a single exon, they can be readily genetically modified with epitope or fluorescent tags [46], or alternatively modified chemically such as via sortase‐mediated labelling [47]. Additionally, due to their elongated CDR3 loop, they are particularly suited for binding concave or cryptic epitopes [45, 48]. One example of such an epitope would be the nanobody Nb80, for which the concave epitope is formed by the intracellular loops of the β_2_‐adrenoceptor [49]. Over the past 10 years, an increasing number of GPCR‐directed nanobodies have been described targeting the extracellular and intracellular regions of the receptor [46]. Although no examples have been specifically described, recent advances in generating synthetic nanobody libraries [50, 51] may prove successful in generating nanobodies with sufficiently long CDR3 loops to access with the ligand binding pocket within the transmembrane domain of GPCRs.

The majority of nanobodies that bind to the extracellular regions of GPCRs target peptide receptors, with recent examples detecting the parathyroid receptor (PTH1R) [52], chemokine CXCR2 receptor [53], and atypical chemokine receptor 3 (ACKR3) [54]. In addition, several nanobodies targeted against extracellular epitopes on the chemokine CXCR4 receptor have been described [55, 56, 57, 58], as well as a panel of i‐bodies: human sdAbs derived from a V_NAR_ scaffold [59, 60]. These studies offer prime recent demonstrations of the utility of sdAbs for endogenous GPCR detection and their use as probes for receptor pharmacology [61]. As an example, members of the Smit group produced a series of nanobodies against CXCR4 [57, 58]. These nanobodies were fused to the Fc domain of a human IgG1 antibody (Nb‐Fc) in order to increase their binding affinity and their ability to perform antibody‐mediated effector function [57]. Using flow cytometry, Nb‐Fc fusions were able to detect endogenous CXCR4 receptors on cells with low (HEK293T), moderate (Jurkat) and high (CCRF‐CEM) levels of expression. One of these CXCR4‐targeted nanobodies was modified with a small peptide tag (HiBiT) for further characterisation of the Nb‐CXCR4 interaction [62]. In the presence of exogenous complementary LgBiT, the HiBiT and LgBiT reconstitute to form the full‐length luciferase NanoLuc and produce luminescence [63]. Using complemented luminescence, nanobody binding was detected in Jurkat cells, which could be displaced by the addition of chemokine receptor ligands [62]. This approach also allowed a comparison of the endogenous CXCR4 expression levels with those of a HEK293 cell line exogenously expressing CXCR4. Griffiths *et al*. [59] produced a panel of ‘i‐bodies’ which were highly specific for CXCR4. One of these (AD‐114) was used to show an altered and increased CXCR4 expression pattern in lung biopsies taken from patients with idiopathic pulmonary fibrosis [60]. These data demonstrate the potential for sdAbs to be used to assess differences in GPCR expression patterns in disease.

Nanobodies have also been used to target endogenously expressed GPCRs on specific cells for photodynamic therapy (PDT). This technique seeks to eradicate tumour cells through the local activation of a photosensitizer with near‐infrared light [64]. Heukers *et al*. [65] generated a nanobody against the viral GPCR US28. US28 is constitutively active in glioblastoma tumours and causes rapid acceleration of glioblastoma progression in a murine *in vivo* model. This nanobody showed high affinity for US28, binding the N terminus and ECL3, and could detect this receptor endogenously expressed in glioblastoma. De Groof *et al*. [66] modified this nanobody with the near‐infrared dye IRDye700DX to determine if nanobody‐targeted PDT was a viable approach to combat glioblastoma. The labelled nanobody accumulated inside US28‐expressing cell spheroids, resulting in a 90% reduction in cell viability in these spheroids upon illumination. Crucially, no change in cell viability was observed in US28‐negative spheroids. Nanobody‐targeted PDT was equally efficacious in both 2D and 3D cell culture models of glioblastoma [66]. This supports the increased tissue penetrance of nanobodies compared to their bulkier conventional antibody counterparts. This study shows the potential for nanobodies targeted against endogenously expressed GPCRs to be used to treat certain diseases.

## Application of genome‐editing approaches to monitor endogenously expressed GPCRs

Fusion of genetically encoded tags to GPCRs has greatly enhanced our understanding of receptor function in live cell models. Using a diverse range of reporter tags, for example fluorescent or bioluminescent proteins or epitope tags, these approaches have been powerful tools to investigate various aspects of GPCR function including their cellular localisation, organisation and signalling. However, due to the technical complexity of engineering the native genome, as well as limitations on the ability to detect lowly expressed proteins, these studies have largely been performed in model cell systems with over‐expressed receptors. Over the last decade, the discovery that endonucleases such as transcription activator‐like effector nucleases (TALENs) and particularly the CRISPR/Cas9 system can be harnessed for site‐specific DNA cleavage has greatly simplified the manipulation of the endogenous genome.

As reviewed previously, CRISPR/Cas9 genome‐engineering has now been widely used to investigate the contribution of different signalling pathways following GPCR activation via knockout of specific signalling effectors or scaffolding proteins [67]. However, these genome‐editing approaches can also be used to knock fluorescent or bioluminescent reporters into the native genome. An advantage of the genome‐editing approaches employed thus far is that only the reporter component is required to be inserted into the genomic locus of the GPCR of interest, rather than the need to add an additional copy of a GPCR with the reporter or the prior knockout of the endogenous receptor. Therefore, with this approach, provided that the GPCR or protein of interest is normally expressed in the cells used, expression of the fusion protein occurs under endogenous promotion and is therefore maintained close to native expression levels. While knock‐ins allow investigation of tagged proteins in their native cellular environments without the need for exogenous expression, only a few studies have used these approaches to detect GPCRs or investigate their function. Of these, most reports have used CRISPR/Cas9 genome editing to insert the luciferase NanoLuc (or small self‐complementing fragments of NanoLuc, NanoBiTs) into the native genome. This approach has allowed native receptor expression to be quantified by luminescence output as well as receptor localisation to be observed by bioluminescence imaging [68, 69]. In part, a key to these studies has been the use of the Nanoluc, which due to its brightness provides the sensitivity to detect low levels of natively expressed proteins. Furthermore, by using CRISPR/Cas9 to fuse NanoLuc to the N terminus of a GPCR, several studies have now used NanoBRET to investigate binding of fluorescent and/or unlabelled ligands [70] to natively expressed GPCRs including genome‐edited adenosine A_2B_ receptors [71], β_2_‐adrenoceptors [68] as well as CXCR4 and ACKR3 chemokine receptors [69]. These NanoBRET ligand binding assays appear particularly suited to genome‐editing approaches as the amount of BRET acceptor, that is a fluorescently tagged ligand, is exogenously applied and easily adjusted. Other aspects of GPCR function previously investigated using over‐expressed GPCRs and NanoBRET or Nanoluc complementation have now also been examined using genome‐edited receptors. This includes monitoring receptor internalisation [68], GPCR‐protein interactions [69] and formation of GPCR‐complexes [72].

In addition to luminescent tags, CRISPR/Cas9 has been used to insert fluorescent tags into the native genome to monitor natively expressed proteins, including those that facilitate GPCR signalling [73], by fluorescence microscopy. An important limitation with this approach is the ability to detect the relatively low levels of endogenous GPCR expression found in most cells without the signal amplification achieved by antibody‐based approaches. However, a recent study [74] using CRISPR/Cas9 genome editing to fuse photo‐switchable fluorescent proteins, mEos3.2, mEos4b, mEGFP or Halotag (subsequently labelled with Janelia Fluor 549) to CXCR4 expressed under endogenous promotion allowed single‐molecule detection and cluster analysis of tagged CXCR4 to be observed using super‐resolution photo‐activated localisation microscopy (PALM). This study also demonstrated improved detection specificity of natively expressed receptors when using fluorescently tagged genome‐edited receptors compared to those labelled with antibodies. They further showed improved sample consistency compared to over‐expressed receptors that allowed for the quantitative measurement of changes in cluster size and distribution on ligand treatment.

These genome‐editing approaches are an important step forward in our ability to detect and monitor natively expressed GPCRs. However, the effect of inserting a tag into the native genome needs to be assessed as this may alter the levels of protein expression. Notably, previous studies have demonstrated small changes in receptor expression, primarily reductions in expression due to the fusion of full‐length NanoLuc to CXCR4 expressed in HEK293 or HeLa cells [69]. Additionally, while not specific to genome‐edited GPCRs, tag‐dependent changes in expression following genome editing have also been noted, with both increases and decreases in expression observed depending on the tag used [69, 74]. However, such changes are orders of magnitude smaller than that seen with over‐expressed receptors; indeed, studies investigating genome‐edited CXCR4 or adenosine A_2B_ receptors found expression to be 40–100‐fold lower than routinely achieved in over‐expressed models [71, 75]. In addition to protein expression, and as with any fusion protein whether genome‐edited or over‐expressed, tagging a GPCR with a reporter component may change its function and needs to be assessed to ensure the relevant observations. While such changes need to be determined empirically, thus far fusion of tags to receptors or proteins that have been validated in over‐expressed models appear to maintain function when expressed under endogenous promotion. Fusion of NanoLuc to the N terminus of CXCR4 maintained ligand binding properties [69], while C‐terminal fusions to CXCR4 displayed the expected signalling properties [74, 75], recruitment of β‐arrestin and internalisation [68, 75]. Similarly, NanoLuc to the N terminus of adenosine A_2B_ receptors maintained ligand binding and signalling [69]. A further consideration of these approaches is that so far, these studies have been performed principally on immortalised cell lines (HEK293, HeLa and PC‐3) that are relatively easy to manipulate via genome editing. It is likely that application of these approaches to primary cells or knock‐in animals will further improve our understanding of GPCRs in their native environment.

## Fluorescent ligands and imaging modalities to study endogenous GPCRs

As one of the inherent properties of a GPCR is to bind a small molecule or peptide, an alternative method to detect endogenously expressed receptors is with fluorescently labelled GPCR ligands. Fluorescent ligands are comprised of an agonist or antagonist for the receptor of interest which is chemically linked to a fluorophore. Their design and application in heterologously expressing systems has been extensively reviewed elsewhere [76, 77]. Fluorescent ligands have been in use since the 1980s to study GPCRs expressed endogenously in cell lines and tissues [78, 79, 80]. Early fluorescent ligands were hampered by high levels of nonspecific binding, dramatic decreases in affinity compared to parent compounds and poor spectral properties for use with the then existing detection methods [81, 82, 83]. Improvements in the rational design of fluorescent ligands [84, 85] and the development of sensitive microscopy technologies have enabled fluorescent ligands to be used to study a number of endogenously expressed GPCRs.

One of the main challenges faced when studying endogenous GPCR expression through imaging modalities is the poor signal to noise ratio [86]. While ongoing developments in fluorescent probe chemistry have provided more selective and brighter ligands, these can still display high nonspecific labelling which adds to the background detection noise. Advanced imaging techniques have been employed to reduce background fluorescence noise alongside granting both high temporal and spatial resolution to decipher GPCR functional dynamics [87, 88, 89]. Highly Inclined and Laminated Optical sheet microscopy (HILO) [90] selectively illuminates only a thin plane as a result of a sharply angled laser which can penetrate ~ 10 µm into the cell. HILO has been employed to study the endogenous thyroid stimulating hormone receptor (TSHR) within primary mouse thyroid cells. Away from the typical model of membrane restricted GPCR signalling, the TSHR has previously been demonstrated to stimulate signalling cascades postinternalisation, but only in over‐expressed systems [91]. By using a fluorescently labelled thyroid stimulating hormone (TSH) and HILO microscopy, agonist‐bound endogenous TSHR was detected at both the plasma membrane and the trans‐Golgi network [88]. The selective illumination of a sharp plane of light to reduce background noise not only allowed detection of low signals typical of endogenous studies but also allowed fast acquisition to capture these real‐time and potentially short‐lived events. The data obtained with HILO, supported by the use of a fluorescently tagged nanobody biosensor, suggested direct activation of G proteins by the TSHR within the trans‐Golgi network, highlighting a physiological role for intracellular signalling [88].

Fluorescence correlation spectroscopy (FCS) is another powerful technique which maximises signal to noise through the measurement of fluorescence fluctuations emanating from a confocal observational volume (~ 0.25 fL). When placed on the cell membrane, the analysis of the fluorescence fluctuations within this volume can inform on receptor organisation and clustering [92]. Endogenously expressed histamine H_1_ receptors in HeLa cells were examined using FCS through recording the fluctuations in the presence of a fluorescent histamine H_1_ receptor antagonist, mepyramine‐BODIPY‐630/650 [87]. Both specific binding to the endogenous histamine H_1_ receptor and nonspecific membrane binding displayed differing diffusion speeds to those recorded in CHO cells stably overexpressing the histamine H_1_ receptor. This suggested a cell type‐specific macromolecular organisation of the receptor. Furthermore, differences between the nonspecific binding measurements suggested there were differences in the organisation of the plasma membrane in these two cell lines. It was postulated that the HeLa plasma membrane environment may be lipid‐raft free, or contained a receptor population which was less constrained than those within the CHO cells. The high temporal resolution of FCS (µs–ms) coupled with selective and bright fluorescent ligands holds the power to interrogate the dynamics and organisation of GPCRs within nanodomains. Using this technique to study endogenous receptor population can avoid forced events and artefacts caused by receptor overexpression [93].

As demonstrated above, fluorescent ligands can be versatile tools to study GPCRs expressed in endogenous systems. Recent improvements in fluorescent ligands with improved subtype selectivity, affinities and physicochemical properties, has made them a valid alternative to antibodies to specifically detect endogenous GPCRs in flow cytometry studies. For example, fluorescently labelled CXCL12 has been used to identify CXCR4 positive T lymphoid SupT1 cells [94]. Additionally, the use of a fluorescent cannabinoid CB2 receptor ligand (NMP6) identified expression of the CB2 receptor on CD4+ T cells [95] which was prevented by preincubation with a CB2 selective agonist. In another example, adenosine A3 receptors were shown to be aggregate in immunomodulatory microdomains on neutrophils using the fluorescent adenosine ligand CA200645 [96]. Fluorescent ligands also have the advantage of allowing the multiplexing of receptor detection alongside quantification of receptor/ligand target engagement at equilibrium in respect to ligand affinity, selectivity, binding kinetics and functional signalling outputs. Flow cytometry has been used to quantify the specific binding of an adenosine A3 receptor agonist (MRS5218) in human promyelocytic leukaemia cells [97] that was displaceable by unlabelled adenosine A3 receptor selective antagonist. Specific binding of fluorescently labelled histamine (BODIPY‐histamine) to the murine histamine H2 or H4 receptor subtypes in Chinese Hamster Ovary (CHO) and murine bone marrow derived mast cells (mBMMCs) has also been shown using flow cytometry and also revealed upregulation of the H4 subtype on mBMMCs in response to immunoglobulin E treatment [98].

A major advantage of using flow cytometry to quantify receptor/ligand engagement is the decreased need for separation of free fluorescent ligand in solution from ligand that is receptor bound, a factor that is critical to other measurements of ligand binding. This is a consequence of the narrow sample volume used so that only a small volume of sample fluid that surrounds the cell is excited. This minimises excitation of unbound fluorescent molecules that are also in solution, diminishing the background fluorescence signal [99]. Sklar *et al*. [80] took advantage of this fact to perform competitive binding kinetic studies of the formyl peptide receptor endogenously expressed on neutrophils. However, the low endogenous expression of GPCRs results in low fluorescence emission levels detected per cell. There are practical limits in flow cytometry for detection of low fluorescence emissions in order to distinguish from cell autofluorescence, although improved instrumentation has aided this distinction [100]. The sensitivity and rapidity of flow cytometry measurements in conjunction with the ability to measure multiple pharmacological parameters simultaneously, therefore makes flow cytometry an attractive method for detecting and characterising the molecular pharmacology of endogenous GPCRs.

Advances in fluorescent ligand design and synthesis, such as the tuning of the physiochemical properties of the linker region to improve ligand affinity and solubility, have made it possible to image GPCRs *in vivo*. An infrared‐emitting α_1_‐adrenoceptor antagonist was designed by Ma *et al*. [101], and used to label endogenous α_1_‐adrenoceptors in *ex vivo* slices of murine prostate tissue. This fluorescent ligand was administered intravenously in mice as a way to image the distribution of α_1_‐adrenoceptors in several tissues. This technique represents a simple and noninvasive way to detect receptor localisation without the need for lengthy and costly RNA‐seq or northern blotting.

Recently, Ast *et al*. [89] described the use of two‐photon microscopy alongside a panel of fluorescently labelled GLP‐1 agonists (LUXendins) to localise endogenous GLP‐1 receptors in mice. These fluorescent ligands displayed exceptional signal‐to‐noise ratios with good affinities for the GLP‐1 receptor. They revealed a distinctive pattern of GLP‐1 receptor expression in murine α‐cells in pancreatic islets, which is important for understanding the action of incretin mimetics in the clinic. Additionally, the good imaging characteristics of these ligands made them amenable to super‐resolution approaches to study subcellular receptor localisations. The GLP‐1 receptors were found to cluster in nanodomains in pancreatic β‐cells, which may have implications for the activation and signalling of these receptors *in vivo*.

Esteoulle *et al*. [102] designed a fluorescent ligand for the oxytocin receptor with a near‐infrared‐emitting dimer moiety. The fluorogenic dimer was designed to be quenched in the aqueous environment of the extracellular medium, but fluoresce strongly in the hydrophobic environment of the lipid bilayer, or when bound to the receptor. Through binding experiments with unlabelled oxytocin antagonists, this fluorescent ligand was found to specifically bind the oxytocin receptor over‐expressed in HEK293 cells [102]. More interesting was the use of this ligand to image murine oxytocin receptors *in vivo* in lactating mice. The ligand showed strong fluorescence in mammary glands, indicative of the presence of oxytocin receptors, with extremely low background fluorescence. The authors suggest this approach could be used for other ligands as a noninvasive method to visualise other GPCRs in their native environment.

## Covalent approaches to label endogenous GPCRs

Fluorescent ligands, by their design, do not react with the receptor of interest and can freely associate and dissociate from the receptor‐binding pocket. This is advantageous when fluorescent ligands are used as probes in ligand binding assays but for studying receptors at endogenous levels, it would be favourable to permanently label the receptor with a fluorophore. Two recent studies have utilised bioorthogonal chemistry to attach a fluorophore to a ligand that is covalently bound to either the adenosine A_2A_ receptor [103] or the cannabinoid CB_2_ receptor [104]. In this two‐step process, first, a ligand with an alkyne handle is covalently bound to the receptor of interest, and then upon photoactivation a fluorescent label is attached to the alkyne handle. This second reaction is classed as a bioorthogonal reaction, which are very selective and do not cross‐react with biological matter present within the experimental set up. The use of bioorthogonal reactions to label GPCRs has the potential to overcome some of the issues of nonspecific binding associated with fluorescent ligands, but to date this has not been investigated. In addition, as the photoaffinity ligand is covalently linked to the receptor of interest, this prevents the addition of subsequent ligands to probe the function of the receptor. Only the photoaffinity probe for the CB_2_ receptor has been used to label endogenously expressed receptors. The CB_2_ receptor is a target for chronic and inflammatory pain [105] and there is a need to determine if the CB_2_ receptor is upregulated in specific immune cell types. With this in mind, the photoaffinity probe was used to investigate the expression of the CB_2_ receptor in peripheral blood mononuclear cells using FACS analysis. The probe detected the highest specific binding in CD19+ B cells which was confirmed by qPCR analysis of CB_2_ receptor mRNA levels. Importantly the probe could distinguish differences in expression levels as for other immune cell types (CD14+ monocytes, CD3+ T cells) both the specific binding of the photoaffinity probe and mRNA levels was lower than in CD19+ cells [104]. Therefore, this photoaffinity ligand has the potential to probe CB_2_ expression levels in different disease states.

An extension of photoaffinity labelling utilises ligand‐directed chemistry to covalently label a receptor of interest without affecting the ligand‐binding site. In ligand‐directed chemistry, a label is connected via a highly reactive, electrophilic linker to a ligand that binds to the receptor of interest. Upon binding of this conjugate, the linker can undergo a substitution reaction with a nucleophilic amino acid side chain, forming a new covalent bond between the label and receptor and consequently separating the ligand from the label [106]. This approach has been successfully applied to label three GPCRs so far; the bradykinin B2 receptor with biotin [107], and the μ opioid and adenosine A_2A_ receptor with a fluorophore [108, 109]. As the ligand is separate from the fluorophore after labelling, it is, in principle, able to freely dissociate from the receptor, and should leave the binding site intact to be probed by additional ligands. This has led ligand‐directed labelling to be known as traceless labelling, and has been shown to be true for the three receptor examples above. An early study which attempted to incorporate a small label into the adenosine A_2A_ receptor in a ligand‐directed manner found that these compounds reduced the number of binding sites available for a radiolabelled ligand, essentially blocking the receptor binding site [110]. Therefore, for each new ligand developed the impact on the ligand binding site will have to be confirmed.

To date, ligand‐directed labelling has been used to label endogenously expressed adenosine A_2A_ receptors and μ opioid receptors. The adenosine A_2A_ receptor is a target for cancer immunotherapy as it is highly expressed on immune cells [111]. Endogenously expressed A_2A_R was detected using FACS analysis on human monocyte‐derived macrophages and visualised on a human breast cancer cell line using confocal microscopy [109]. The ligand‐directed label described for the μ opioid receptor has been used to map opioid‐sensitive neurons in rat and mouse brains. Due to the function of the receptor being preserved after labelling, agonist‐mediated internalisation was visualised in locus coeruleus neurons [108] and understanding the expression pattern and responsiveness may aid the development of safer analgesics that target this receptor [112]. Ligand‐directed labelling of GPCRs offers a noninvasive approach to visualise receptors and opens up a huge number of possibilities to study ligand binding, receptor trafficking and signalling in endogenously and clinically relevant systems.

## Summary and future directions

The approaches discussed above demonstrate the rapid advances being made to detect endogenous GPCRs in their native environment. Improvements in fluorescent ligand design will result in more studies describing the subcellular GPCR localisation and how this may change in response to cellular stress. These approaches could also be used in conjunction with advanced microscopy (Table 2), such as single‐molecule tracking or FCS, to probe the role of GPCR dimerisation and organisation into higher order oligomers in their native environment. Additionally, the recent studies using fluorescent ligands *in vivo* are particularly exciting, as these demonstrate the possibility to investigate receptor distribution in disease models.

**Table 2:** Current and evolving microscopy techniques for studies with GPCRs expressed at endogenous levels. Advantages and disadvantages for each technique are shown, alongside examples and future applications which could be applied to the investigation of endogenous receptors.

Technique	Advantages	Disadvantages	Example GPCRs	Potential future applications or related techniques
FCS	Quantify concentration and diffusion characteristics of fluorescent species High temporal resolution Modelling statistics improve as concentration of fluorescent species decreases	Low throughput Cell membrane recordings are technically challenging Only the average concentration/diffusion coefficient of a population can be described	Histamine H_1_ [87]	FCCS – Fluorescence Cross‐Correlation Spectroscopy. Separate and combined diffusion properties of two different fluorophores can be resolved allowing investigation of protein–ligand and protein–protein interactions, e.g. dimerisation. Scanning FCS – Observation volume is scanned repeatedly across the sample to record diffusion at multiple locations
HILO	Thin imaging plane penetrates ~ 10 µm into cell Reduced background fluorescence increases signal to noise Single‐molecule imaging possible	Limited field of view Cannot penetrate deep samples	TSHR [88]	TIRF – Total Internal Reflection Fluorescence microscopy. Evanescent wave only excites fluorophores within ~ 100–200 nm of coverslip surface ideal to study membrane localised proteins. LSFM – Light Sheet Fluorescence Microscopy. The field of view is imaged perpendicular to a thin sheet of laser excitation. This leads to reduced background from out of focus fluorescence, increased signal‐to‐noise and faster acquisition. Much larger sample, including whole embryos, can be imaged
Bioluminescence Imaging	No photo‐toxicity nor photo‐bleaching No specialised buffers or complicated workflow	Requires bright luciferase (e.g. NanoLuc) Requires very sensitive camera, e.g. Cooled EMCCD Longer exposure times required (10–60s) for endogenous levels	CXCR4 [69]	BRET imaging – Imaging of the resonance energy transfer from luciferase to fluorescent protein or ligand linked fluorophore can enable the study of protein–protein interaction or ligand binding respectively
Confocal/widefield microscopy	Can be performed without additional specialised microscopy equipment Compatible with both fixed and live samples and almost all probes	Low signal to noise Can be limited in spatial resolution Photo‐bleaching and photo‐toxicity from long exposure times required to detect at endogenous levels	Adenosine A_2A_ receptor [109] GLP‐1 receptor [89] µ opioid receptor [108]	Super‐resolution microscopy. Multiple applications are now possible which allow capture of single‐molecule localisation and high spatial resolution (See below). FRET – Forster Resonance Energy Transfer. Protein–protein and protein–ligand interactions can be detected through measuring the fluorescence of a donor and acceptor fluorophore
Super‐resolution techniques, e.g. PALM, STED (Stimulated Emission Depletion Microscopy)	High spatial resolution Single‐molecule imaging possible Improved localisation microscopy	Sample prep and buffer selection may require optimisation (PALM) Higher laser powers may be required Requires specific fluorophore characteristics	CXCR4 [74] GLP‐1 receptor [89]	STORM – STochastic Optical Reconstruction Microscopy. Reconstruction of stochastically activated photo‐switchable fluorophores to detail precise localisation data. Could potentially utilise fluorescently tagged GPCR antibodies or nanobodies. Expansion Microscopy. Physical enlargement of a specimen attached to a polymer to allow nanoscale imaging with a standard confocal microscope

GPCRs are now known to be capable of signalling from intracellular compartments, such as from the endosome or trans‐Golgi network [88, 113, 114]; however, only a few studies have shown this to be the case for endogenously expressed GPCRs. This is important as the trafficking and organisation of GPCRs can change depending on the expression level [115]. The techniques discussed above could expand on this knowledge to determine if this compartmentalised signalling occurs for a subset of GPCRs, or is a widely occurring phenomenon. CRISPR/Cas9 offers the ability to introduce disease‐relevant single nucleotide polymorphisms (SNPs) and study their effects in relevant cellular or animal models. However, at the time of writing few studies have used this approach, with examples including the β_1_‐adrenoceptor [116], and the orphan receptor GPRC6A [117]. Given the variation of the GPCR repertoire in a patient population [118], understanding the role SNPs have on endogenous receptor expression and function could offer important targets for personalised medicine. Additionally, the combination of CRISPR/Cas9 and FRET/BRET techniques offers the potential to study ligand–receptor interactions at endogenous levels in primary cells. Studying endogenously expressed GPCRs in native conditions could lead to the development of novel biomarkers or drug targets previously overlooked.

## Conflict of interest

The authors declare no conflict of interest.

## Author contributions

MS, LAS, CWW, LEK, JG, SJB and SJH all contributed to the planning and writing of the manuscript.
